# Microbial abundance in surface ice on the Greenland Ice Sheet

**DOI:** 10.3389/fmicb.2015.00225

**Published:** 2015-03-24

**Authors:** Marek Stibal, Erkin Gözdereliler, Karen A. Cameron, Jason E. Box, Ian T. Stevens, Jarishma K. Gokul, Morten Schostag, Jakub D. Zarsky, Arwyn Edwards, Tristram D. L. Irvine-Fynn, Carsten S. Jacobsen

**Affiliations:** ^1^Geological Survey of Denmark and GreenlandCopenhagen, Denmark; ^2^Center for Permafrost, University of CopenhagenCopenhagen, Denmark; ^3^Department of Ecology, Charles University in PraguePrague, Czech Republic; ^4^Centre for Glaciology, Aberystwyth UniversityAberystwyth, UK; ^5^Centre for Polar Ecology, University of South BohemiaČeské Budějovice, Czech Republic; ^6^Department of Plant and Environmental Sciences, University of CopenhagenCopenhagen, Denmark

**Keywords:** glacier ice, microbial abundance, Greenland Ice Sheet, epifluorescence microscopy, flow cytometry, quantitative PCR, multivariate analysis

## Abstract

Measuring microbial abundance in glacier ice and identifying its controls is essential for a better understanding and quantification of biogeochemical processes in glacial ecosystems. However, cell enumeration of glacier ice samples is challenging due to typically low cell numbers and the presence of interfering mineral particles. We quantified for the first time the abundance of microbial cells in surface ice from geographically distinct sites on the Greenland Ice Sheet (GrIS), using three enumeration methods: epifluorescence microscopy (EFM), flow cytometry (FCM), and quantitative polymerase chain reaction (qPCR). In addition, we reviewed published data on microbial abundance in glacier ice and tested the three methods on artificial ice samples of realistic cell (10^2^–10^7^ cells ml^−1^) and mineral particle (0.1–100 mg ml^−1^) concentrations, simulating a range of glacial ice types, from clean subsurface ice to surface ice to sediment-laden basal ice. We then used multivariate statistical analysis to identify factors responsible for the variation in microbial abundance on the ice sheet. EFM gave the most accurate and reproducible results of the tested methodologies, and was therefore selected as the most suitable technique for cell enumeration of ice containing dust. Cell numbers in surface ice samples, determined by EFM, ranged from ~ 2 × 10^3^ to ~ 2 × 10^6^ cells ml^−1^ while dust concentrations ranged from 0.01 to 2 mg ml^−1^. The lowest abundances were found in ice sampled from the accumulation area of the ice sheet and in samples affected by fresh snow; these samples may be considered as a reference point of the cell abundance of precipitants that are deposited on the ice sheet surface. Dust content was the most significant variable to explain the variation in the abundance data, which suggests a direct association between deposited dust particles and cells and/or by their provision of limited nutrients to microbial communities on the GrIS.

## Introduction

Glaciers and ice sheets cover 10% of Earth's land area and contain distinct microbe-dominated ecosystems that are highly sensitive to climate warming (see Hodson et al., 2008; Anesio and Laybourn-Parry, 2012, for reviews). Microbes in glacial ecosystems play important roles in local and regional biogeochemical cycling processes (e.g., Foreman et al., 2007; Hodson et al., 2007; Stibal et al., 2008a; Anesio et al., 2010; Telling et al., 2012) and may contribute to glacier ice melting (Takeuchi et al., 2001; Yallop et al., 2012). Measuring microbial abundance in glacier ice and researching its spatiotemporal variability and its controls is necessary to estimate microbial growth and activity, as well as to estimate carbon stocks and flows in glacial ecosystems, and future extrapolations of these measurements are essential for the prediction of microbial responses to changes in climate and anthropogenic influences in the cryosphere (Stibal et al., 2012a).

Our knowledge of microbial abundance in glacier ecosystems is, however, sketchy in comparison with other ecosystems (Whitman et al., 1998), since it is based on a low number of samples from accessible glacier sites. As a result, our understanding of the factors controlling microbial abundance in the ice is limited and current large-scale estimates of microbial biomass in glacier ice are empirical and span many orders of magnitude. For example, a recent study offered a first-order estimate of between 10^25^ and 10^29^ microbial cells entombed in glacier ice world-wide, and emphasized that elevated biomass is associated with glacier surfaces and beds (Irvine-Fynn and Edwards, 2014). Moreover, glacier ice tends to have a low microbial abundance, and the microbial cells are typically mixed with or attached to mineral particles. This poses a challenge for the cell enumeration of most glacier samples, including cryoconite (surface debris), which is a conglomerate of mineral particles, microbial cells and organic matter (Hodson et al., 201b; Langford et al., 2010), and sediment-laden basal ice (Foght et al., 2004; Yde et al., 2010; Montross et al., 2014).

Traditionally, epifluorescence microscopy (EFM) has been used to enumerate microbial cells in aqueous and sediment samples, including glacier ice, and sediments (Karl et al., 1999; Priscu et al., 1999; Abyzov et al., 2001; Säwström et al., 2002). This method is labor-intensive and slow compared to flow cytometry (FCM), which has previously been used for glacier ice microbial abundance analysis (Karl et al., 1999; Yao et al., 2008; Miteva et al., 2009; An et al., 2010; Irvine-Fynn et al., 2012). However, FCM is sensitive to higher particulate loads, which may result in instrumentation blockages (Vesey et al., 1994) and underestimations due to cell adhesion to abiotic particles (Amalfitano and Fazi, 2008). Recently, quantitative PCR (qPCR) has gained popularity in glacier ecology studies as it allows for a combined analysis of microbial abundance and diversity from the same nucleic acid extract (Hamilton et al., 2013; Zarsky et al., 2013; Stibal et al., 2015). However, due to differing numbers of gene copies in each microbial species (Klappenbach et al., 2001), and the efficacy of nucleic acid extraction (Krsek and Wellington, 1999) and amplification techniques (Lindberg et al., 2007; Albers et al., 2013), caution must be exercised when converting qPCR results into cell numbers.

The Greenland Ice Sheet (GrIS) is the largest ice body in the northern hemisphere and hosts Earth's largest seasonally melting glacier surface ice ecosystem (>200,000 km^2^ and expanding; Hodson et al., 2010a; Fettweis et al., 2011). Microbial abundance, diversity, and activity in snow and cryoconite in some portions on the GrIS have been found to vary with distance from ice-free land. This variability has been attributed to differences in environmental disturbances, sources of microbial inocula and nutrients, and melt season duration (Hodson et al., 2010a; Stibal et al., 2010, 2012b, 2015; Telling et al., 2012; Cameron et al., 2015). However, there are currently no data on microbial abundance in bare ice, the dominant supraglacial environment in terms of volume and area, and the factors that control it.

The aim of this paper is to quantify for the first time the abundance of microbial cells in surface ice from geographically distinct sites on the GrIS and to identify factors responsible for its variation. In order to obtain robust cell numbers, we tested all three common methods of microbial enumeration (EFM, FCM, qPCR) on artificial ice samples of known, and realistic, cell and mineral particle concentrations prior to analysis of our samples. We then used multivariate statistical analysis to test the significance of environmental characteristics and reviewed the published data on microbial abundance in glacier ice in order to put our results into context.

## Materials and methods

### Sample collection

**Figure 1:**
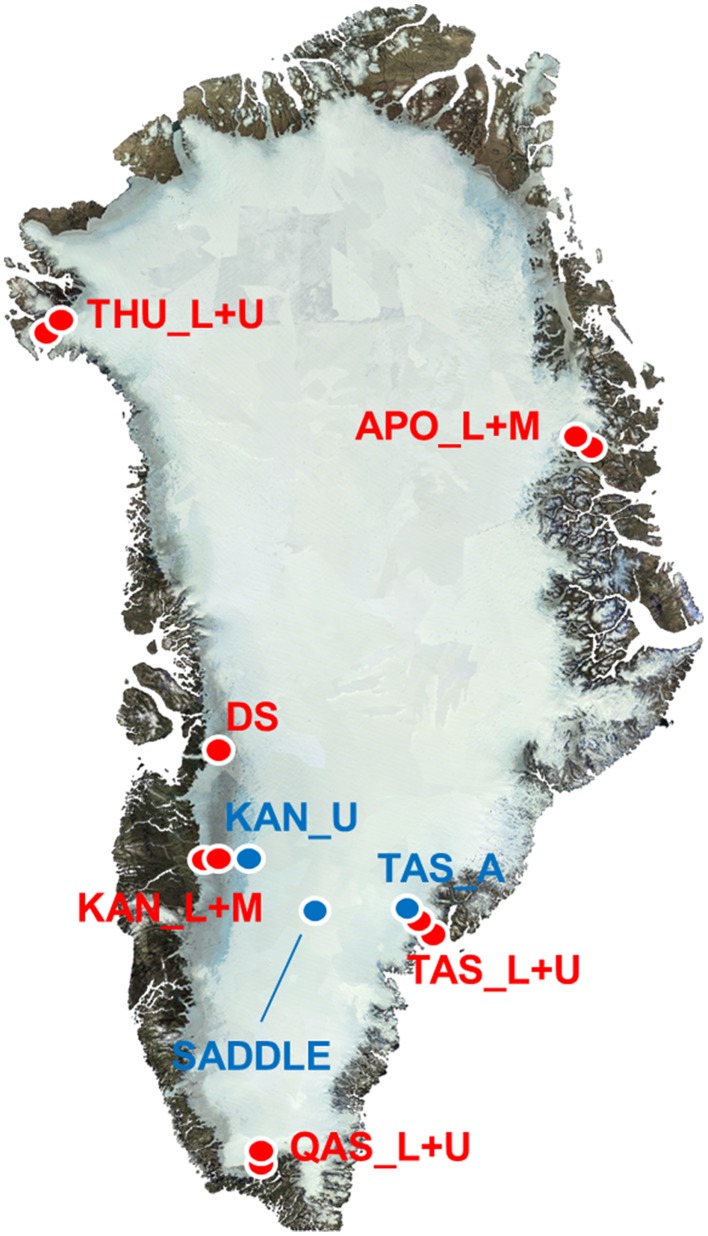
**Map of sampling sites on the GrIS**. Red dots represent sites in the ablation area and blue dots in the accumulation area of the ice sheet. The background clear sky end of melt season mosaic is by J. E. Box using MODIS data from year 2011.

Samples of Greenland surface ice were collected from the ice sheet and an isolated ice cap between May and September 2013 from 14 sites at 7 geographically distinct locations (Table 1, Figure 1). Most sites were in the vicinity of an established meteorological station of the PROMICE network (http://promice.org) and were named after the nearest settlement or geographical feature (THU, Thule; KAN, Kangerlussuaq; QAS, Qasimiut; TAS, Tasiilaq; APO, A. P. Olsen ice cap). Additional samples of surface ice were taken at “Dark Site” (DS), one of the darkest 5 km pixels in optical satellite imagery after Box et al. (2012), and at a site situated in the accumulation area near the topographical divide of the southern ice sheet (Saddle). The sites were characterized by their geographical position (the N and W coordinates) and altitude which were measured by a hand-held GPS, surface type (bare ice vs. multi year snow a.k.a. firn), and distance from the nearest ice-free land determined in Google Earth using the distance tool with a precision of 0.5 km. The regional climate model HIRHAM5 was used to obtain additional climate data for each site. This model provides realistic simulations of the climate over Greenland, which are validated against observations from meteorological stations at the coast and on the ice sheet (Lucas-Picher et al., 2012). The data obtained from the model included the number of days with a positive surface air temperature and a positive surface energy balance (“melt days”) from the beginning of the year until the day of sampling, and the time elapsed from the last snowfall event at the moment of sampling (Table 1).

**Table 1:** **Description of 2013 sampling sites at the surface of the GrIS**.

**Site name**	**Position**	**Distance to ice-free land (km)**	**Altitude (m)**	**Surface type**	**Date (DOY)**	**Days of T_s_ > 0°C in 2013**	**Melt days in 2013**	**Days since snow**
THU_L	76°23.991′N 68°15.921′W	1.5	570	ice	12 Aug (224)	46	n.a.	11
THU_U	76°25.181′N 68°8.706′W	3	770	firn	13 Aug (225)	40	134	11
DS	69°28.56′N 49°34.838′W	18	956	ice	25 Jun (176)	15	n.a.	2
KAN_L	67°5.798′N 49°56.303′W	5	680	ice^**^	19 Sept (262)	90	189	7
KAN_M	67°3.964′N 48°49.356′W	42	1270	ice^**^	19 Sept (262)	38	158	2
KAN_U	67°0.014′N 47°1.162′W	112	1850	firn^**^	22 Sept (265)	9	190	5
QAS_L	61°1.873′N 46°50.91′W	1.5	310	ice	20 Aug (232)	124	164	47
QAS_U	61°10.653′N 46°49.042′W	12	890	ice^**^	20 Aug (232)	65	182	41
TAS_L	65°38.46′N 38°53.895′W	1.5	270	ice	27 Aug (239)	108	165	62
TAS_U	65°41.975′N 38°51.995′W	5	580	ice	29 Aug (241)	88	184	1
TAS_A	65°46.864′N 38°54.193′W	10	891	firn^**^	27 Aug (239)	68	n.a.	1
APO_L	74°37.471′N 21°22.507′W	0.5	644	ice^***^	1 May (121)	0	n.a.	1
APO_M	74°38.634′N 21°28.110′W	0.5	874	ice^***^	1 May (121)	0	n.a.	1
SADDLE	66°0.033′N 44°30.083′W	180/230^*^	2460	firn	8 Jul (189)	0	n.a.	1

*DOY, day of year 2013; T_s_, surface air temperature; n.a., data not available*.

* *Site is ca 180 km from the eastern edge and 230 km from the western edge of the ice sheet*.

** *Samples may have been affected by fresh snow due to high wind during sampling*.

*** *Samples may have been contaminated due to breakdown of drilling equipment and additional handling*.

Ten surface ice cores (~15 cm long) were extracted at each site, except Saddle, using a small handheld drill and custom-built stainless steel corers (~20 cm^2^ surface area). The corers were autoclaved and kept sterile in polypropylene bags prior to use. The ice cores were transferred to sterile 750 ml WhirlPak bags (Nasco, USA). At Saddle, samples were obtained using a 9 cm diameter Kovacs coring drill, using sterile autoclaved corers. A deeper (220 cm) surface ice core was extracted at Saddle and cut into sections 10–30 cm long. Saddle samples were taken in order to compare microbial cell numbers of winter snow layers, from 2012 to 2013, with the summer 2012 refrozen melt layer (Nghiem et al., 2012). All samples were kept frozen in insulated boxes until transportation to Copenhagen where samples were stored at −20°C until analysis.

### Cell count method testing

Artificial ice samples were prepared using deionized water, quartz dust, and a culture of *Delftia acidovorans* in order to simulate glacier ice containing different amounts of debris and microbial cells. *Delftia* is a genus of Betaproteobacteria often found in glacial environments including surface ice (Zeng et al., 2013), cryoconite (Stibal et al., 2015), and basal ice (Skidmore et al., 2005). The water used (MilliQ, Millipore, USA) was checked for microbial cells using EFM (see below). Quartz dust (2600 mg ml^−1^, particle size <63 μm; Sigma-Aldrich, Germany) was furnaced at 550°C for 5 h prior to use. The cell abundance of the *D. acidovorans* culture used was determined by EFM immediately before preparing the artificial ice samples. The dust concentrations used were from 0.1 to 100 mg ml^−1^, and the cell concentrations used ranged from 10^2^ to 10^7^ cells ml^−1^, resulting in cell:dust ratios between 1 and 10^8^ cells mg^−1^, roughly equivalent to 0.2–20,000,000 cells per dust particle. Samples containing no cells and/or no dust were tested in parallel.

Accuracy *A* was quantified as

$$A=1-\left(\frac{\left|\bar{X_{m}}-X_{e}\right|}{X_{e}}\right)$$

where *X_e_* is the expected abundance and *X_m_* the measured value. *A* can range between 1 (100% accuracy) and 0 (no cells or twice as many as expected), and it can assume negative values when the measured abundance is more than twice as high as the expected value; however, for better plot clarity negative *A* values were manually corrected to 0. Standard deviations of triplicate measurements, representing the reproducibility of the analyses, were calculated and expressed as percentage of mean; values >100% were manually corrected to 100% for better clarity in the contour plots.

### Greenland ice sample analysis

Prior to analysis, ice samples from each location were pooled together and placed in a pre-furnaced (550°C for 5 h), foil-covered beaker and allowed to melt at 4°C. After melting, subsamples for EFM (150 ml) and FCM (15 ml) were taken. EFM enumerations were conducted immediately after subsampling, whereas samples for FCM were fixed with paraformaldehyde (final concentration 2%) and stored at 5°C until analysis. From the remaining sample, 300 ml was filtered through Sterivex GP 0.22 μm polyethersulfone filters (Millipore, USA) into acid washed Duran bottles. The filters were subsequently used for DNA extraction, while the filtered water was used for physico–chemical analysis. pH and electrical conductivity (EC) were measured using a Multi 3430 multimeter with a SenTix 940 pH electrode and a TetraCon 925 conductivity cell (WTW, Germany). Dissolved organic carbon (DOC) and total dissolved nitrogen (TDN) were measured on a TOC-V_CPH_ analyzer with a TNM-1 nitrogen unit (Shimadzu, Japan). Nitrate (NO^−^_3_) and phosphate (PO^3−^_4_) were analyzed by ion chromatography (IC) using an IonPac AS 14 column (Dionex, USA). Ammonium (NH^+^_4_) was determined on a Fiastar 5000 analyzer (Gerber Instruments, Switzerland). The detection limits, calculated as 3 standard deviations of procedural blanks, were 1.17 mg l^−1^ for DOC, 0.20 mg l^−1^ for TDN, and 4.4 μg l^−1^ for NH^+^_4_. No NO^−^_3_ or PO^3−^_4_ were detected in the procedural blanks and so 0.05 and 0.025 mg l^−1^ were assumed to be the detection limits for nitrate and phosphate, respectively, determined by previous testing. The remainder of the sample was filtered through a pre-weighed GF/F 0.7 μm glass fiber filter (Whatman, UK) in order to determine the dust load. The filter papers were then dried at 105°C for 5 h and re-weighed, and the amount of dust normalized to filtrate volume.

Samples were analyzed by EFM after staining with the DNA stain acridine orange (AO). 10 ml of sample was filtered onto a sterile 0.2 μm MontaMil black polycarbonate filter (Frisenette, Denmark). Dried filters were placed in a Petri dish containing AO (0.04% final concentration; Fluka, Switzerland) for 2 min, then in deionized water for another 2 min, dried and mounted on microscopic slides with immersion oil. More than 300 AO-stained cells were enumerated on each slide with an Olympus BX50 epifluorescence microscope (Olympus Optical, Japan) using the filter block U-N31001 (Chroma Technology, USA). Blanks with no cells were counted in parallel.

For FCM, samples were analyzed using a SH-800-EC cell sorter (Sony Biotechnology, Japan) according to protocols optimized for supraglacial meltwater. All samples were vortexed on a Vortex-Genie 2 (Cambio, UK) for 30 s before each stage of processing. Field samples and most artificial ice samples were analyzed undiluted while artificial ice samples with dust concentrations of 10 and 100 mg ml^−1^ were diluted 10- and 100- fold with 0.1 μm filtered deionized water, respectively, to prevent potential blockage of the cell sorter sample tubing. To control for autofluorescence and dust background, stained and unstained aliquots were processed in parallel. For stained samples, 2 μl of 10,000× SYBR Gold (in DMSO; Life Technologies, UK) stock solution was diluted to 1 ml in phosphate buffered saline (pH 7.4), and 1 μl of this solution was used to stain 2 ml of sample for 30 min at room temperature (~23°C) prior to analysis. The cell sorter was operated with samples interrogated at a flow rate of 21 μl min^−1^ for 30 s with 488 nm laser excitation and fluorescence emissions in the 520–550 nm channel measured along with forward and back scatter. Populations were gated manually.

DNA was extracted from the Sterivex filters using the PowerWater Sterivex DNA Isolation Kit (MO BIO Laboratories, USA), following the manufacturer's protocol. An unused Sterivex filter was extracted alongside the samples as a procedural control. Quantitative PCR of 16S rRNA genes was performed using a CFX96 Touch system (Bio-Rad, USA). Reaction mixtures (20 μl total) consisted of 1 μl of template DNA, 10 μl of SYBR Premix DimerEraser (TaKaRa, Japan), and 0.6 μl of forward and reverse primers (10 pmol μl^−1^). The primers used were 341F (5′-CCTACGGGAGGCAGCAG-3′) and 518R (5′-ATTACCGCGGCTGCTGG-3′). The cycle program was 95°C for 30 s followed by 50 cycles of 95°C for 30 s, 55°C for 30 s, and 72°C for 30 s. The reaction was completed by a final 72°C elongation step for 6 min and followed by high-resolution melt curve analysis in 0.5°C increments from 72 to 98°C. All qPCR reactions were performed in triplicate and were prepared under DNA free conditions in a pressurized clean-lab with a HEPA filtered air inlet and nightly UV-irradiation. Standards of bacterial 16S rRNA genes were prepared by extracting DNA from a serially diluted culture of *E. coli*. The gene copy number of the highest standard was 1.12 × 10^7^ μl^−1^. The detection limits were 1.6 × 10^2^ and 1.7 × 10^3^ gene copies per μl of reaction volume for the artificial ice samples and the Greenland ice samples, respectively. Due to the much diluted nature of our samples, potential inhibition due to humic acid or other inhibitory compounds was considered unlikely and was not evaluated.

### Statistical analysis

Multivariate statistical analysis was used to explain the variation in the data, as described previously (Stibal et al., 2012b). All nutrient concentration and microbial abundance data were log transformed prior to analysis and all data were standardized and centered. Data below detection limit (b.d.) were treated as zeroes. Redundancy analysis (RDA) with interactive forward selection and 999 Monte Carlo permutations in an unrestricted mode was used to explain the variation in the data. The *p*-values were corrected for multiple testing using false discovery rate. All the analyses were performed in the multivariate data analysis software Canoco 5 (ter Braak and Šmilauer, 2012).

## Results and discussion

### Microbial cell enumeration testing

Accurate enumeration of microbial cells in glacial samples with high debris contents is notoriously difficult due to the problems associated with masking by debris and the difficulty in obtaining adequate sample volumes (Foght et al., 2004; Langford et al., 2010; Hodson et al., 2013). The results of our artificial ice abundance measurements are illustrated in Supplementary Figure S1. EFM gave the highest accuracies of the three methods tested (up to 0.97), as well as the best reproducibility (standard deviation down to 1.2% of mean). However, the accuracy values within the realistic ranges of cell and dust concentrations were still low (between 0.15 and 0.23), and were only higher (>0.75) in the samples with more than 10^4^ cells ml^−1^ and without dust addition. In contrast, both FCM and qPCR performed poorly, with all accuracy values below 0.7, even in samples with no dust added, and poor reproducibility (Supplementary Figure S1). No significant correlations between the FCM and qPCR data, expressed as the percentage of the respective EFM values and dust concentrations, were found (data not shown). We acknowledge a potential bias in favor of EFM since the expected values (*X_e_* in Equation 1) were determined by this method; however, this bias is probably small due to the high reproducibility of cell enumeration by EFM in a high-abundance and dust-free bacterial culture.

FCM has a good history of application to glacier samples (Karl et al., 1999; Yao et al., 2008; Miteva et al., 2009; An et al., 2010; Irvine-Fynn et al., 2012); however, in this study its performance was suboptimal relative to EFM. Three factors may account for this. First, interference from dust particles is prominent. While concurrent analysis of unstained samples has been sufficient to mitigate against dust interference in supraglacial meltwater (Irvine-Fynn et al., 2012), the higher sediment loads which may be found in glacier ice may complicate analyses and result in enumeration of undesirable “noise” particles, adsorption of cells to dust particles or spurious abiotic autofluorescence. Second, the number of cells analyzed per sample under the typical flow rates and parameters used is small. This may compromise the accuracy of counts. Third, the cell sorter used is unable to measure side scatter, the preferred metric for the identification of individual cell “events” (Irvine-Fynn et al., 2012). Use of forward and back scatter may explain the underestimation of cell counts in this study (Table 3), as cells adsorbed to dust particles or other cells are only recorded as a single event. However, the inter-replicate reproducibility of FCM was relatively good. It is clear that to realize the potential of FCM in high-throughput robust enumeration of cells against higher backgrounds of dust levels in glacier ice (Irvine-Fynn and Edwards, 2014), further work to optimally deconvolve dust and cell populations is necessary. Detaching cells from mineral particles may be required prior to analysis, even though these techniques may only yield 80–90% efficiency (Amalfitano and Fazi, 2008).

While PCR is a useful tool in diversity studies, its suitability for accurate quantification of cells in natural microbial communities is limited by various biases. The fact that no correlation was found between the qPCR/EFM abundance ratios and the concentration of dust, the most likely source of potentially inhibitory compounds, suggests that inhibition of PCR polymerases (Lindberg et al., 2007; Albers et al., 2013) was not a significant bias in the analysis of our ice samples. However, other biases may have been at play, such as differential extraction efficiencies for different microbial groups (Krsek and Wellington, 1999) and different numbers of the ribosomal RNA operon copies per cell (Klappenbach et al., 2001) Therefore, based on our results, traditional EFM is recommended when accurate numbers of microbial cells in ice samples containing dust particles are required, despite its laboriousness. Caution must still be exercised not to overinterpret differences in abundance within an order of magnitude.

### Physico–chemical characteristics of greenland surface ice

Physico–chemical characteristics of the melted ice from the surface of the GrIS are shown in Table 2. The dust load measured by the filtration method was variable (0.01–1.9 g dust per liter of melted ice, mean ± sd: 0.39 ± 0.51 g l^−1^), and consistent with particle concentrations measured by FCM (39–474 particles per ml; 150 ± 152 ml^−1^), with two exceptions (QAS_L, APO_L; Table 2). The highest dust load was detected in samples from DS, QAS_L, and APO_M while samples from the Kangerlussuaq transect (KAN_L/M/U) were lowest in dust. Electrical conductivity ranged between 1.7 and 4.1 μS cm^−1^ and pH ranged between 5.40 and 5.97, with no obvious trends in the samples. DOC ranged from <1.17 to 3 mg l^−1^ while TDN was below the detection limit of 0.2 mg l^−1^ in all the samples. Ammonium concentrations were between <4.4 and 25 μg l^−1^, while those of nitrate ranged between <0.05 and 0.09 mg l^−1^. Phosphate concentrations were below the detection limit of 0.025 mg l^−1^ in all the samples measured. The nutrient concentrations were similar to those previously measured in surface ice on the GrIS (Telling et al., 2012) and within the range reported from other glaciers (Tranter et al., 2004; Bagshaw et al., 2007; Hodson et al., 2013).

**Table 2:** **Physico–chemical characteristics of surface ice sampled on the GrIS**.

**Site**	**Dust**		**EC μS cm^−1^**	**pH**	**Nutrient concentrations**		
	**g l^−1^**	**10^3^ particles ml^−1^**			**DOC mg l^−1^**	**NO^−^_3_ mg l^−1^**	**NH^+^_4_ μg l^−1^**
THU_L	0.10	70±3.4	4.0 ± 0.38	5.97 ± 0.09	3.0±1.4	0.05 ± 0.05	25±0.93
THU_U	0.29	81±13	2.5 ± 0.25	5.64 ± 0.20	1.6±0.55	b.d.	23±32
DS	0.51	146±38	2.8 ± 0.06	5.78 ± 0.09	1.1±0.39^*^	0.02 ± 0.04^*^	18±16
KAN_L	0.03	67±34	2.0 ± 0.12	5.83 ± 0.03	b.d.	0.07 ± 0.02	b.d.
KAN_M	0.08	47±10	2.0 ± 0.15	5.62 ± 0.08	0.36±0.32^*^	0.06 ± 0.00	6.5±5.7
KAN_U	0.01	39±5.7	1.9 ± 0.10	5.40 ± 0.04	b.d.	0.09 ± 0.03	b.d.
QAS_L	0.93	425±154	2.0 ± 0.10	5.82 ± 0.05	2.7±0.51	b.d.	b.d.
QAS_U	0.20	62±42	2.0 ± 0.15	5.65 ± 0.04	2.3±1.1	b.d.	7.9±3.3
TAS_L	0.35	122±50	1.9 ± 0.30	5.59 ± 0.19	b.d.	b.d.	7.7±13
TAS_U	0.18	62±17	1.7 ± 0.06	5.63 ± 0.03	b.d.	b.d.	4.7±4.5
TAS_A	0.20	46±11	2.4 ± 0.06	5.69 ± 0.08	b.d.	b.d.	23±13
APO_L	0.36	474±103	4.0 ± 0.12	5.73 ± 0.05	1.2±0.72	b.d.	4.7±8.1
APO_M	1.87	317±53	4.1 ± 0.46	5.53 ± 0.01	1.3±0.74	b.d.	3.7±6.4^*^

*Mean ± st.dev.; n = 3, except dust weight (n = 1); b.d., below detection limit*.

* *Values below detection limit were treated as zeroes so the mean values shown can be below the respective detection limits*.

### Microbial abundance in greenland surface ice

Microbial abundances in the surface ice samples from the GrIS were measured by the three methodologies (Table 3). Cell numbers within ice samples, determined by EFM, spanned three orders of magnitude (from ~ 2 × 10^3^ to ~ 2 × 10^6^ cells ml^−1^). The FCM analysis resulted in lower cell numbers (1.5 – 65% EFM) in all cases except one (THU_U; 400%). The 16S rRNA gene copy numbers determined by qPCR produced values of the same order of magnitude as those measured by EFM, assuming 5–10 copies per cell, except for the DS and TAS samples where the qPCR values were an order of magnitude higher than those determined by EFM (Table 3). The highest cell numbers were determined in samples from QAS_L, TAS_L, DS, and APO. Unlike in the first three samples, the high abundances in the APO samples were unexpected due to the early sampling date and the fact that no liquid water was present in the surface ice during sampling. Two possible explanations for this result are, first, contamination due to a breakdown of the drilling equipment and the necessity to handle the ice samples in a non-sterile way, and second, the high dust content (Table 2).

**Table 3:** **Microbial cell/16S rRNA gene copy abundances in the surface ice samples from the GrIS determined by epifluorescence microscopy (EFM), flow cytometry (FCM), and quantitative PCR (qPCR)**.

**Site**	**Microbial abundance**		
	**EFM (10^3^ cells ml^−1^)**	**FCM (10^3^ cells ml^−1^)**	**qPCR (10^5^ copies ml^−1^)**
THU_L	34±12	22±7.9	4.5±1.3
THU_U	3.7±0.29	15±16	0.46±0.39
DS	370±38	5.8±3.9	200±8.7
KAN_L	3.1±0.74	0.60±0.40	0.24±0.11
KAN_M	28±5.2	b.d.	8.8±3.0
KAN_U	1.9±1.2	0.19±0.44	0.29±0.14
QAS_L	1300±82	26±22	260±120
QAS_U	110±1.8	1.8±0.07	22±6.2
TAS_L	260±83	5.3±2.1	140±5.3
TAS_U	74±8.1	13±12	63±43
TAS_A	16±2.5	1.2±0.58	17±2.2
APO_L	560±39	71±77	110±24
APO_M	1900±350	28±17	240±78

*Mean ± st.dev.; n = 3; b.d., below detection limit*.

**Figure 2:**
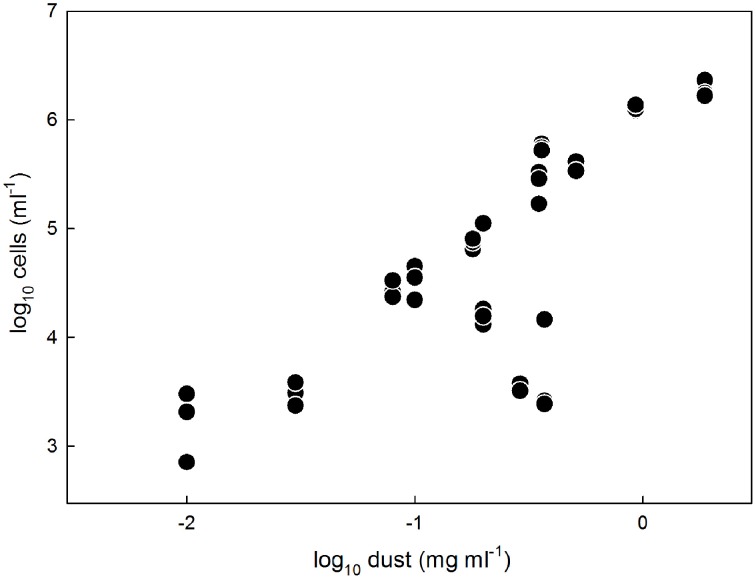
**Microbial cell abundances in surface ice samples from the GrIS determined by EFM plotted against the respective dust concentrations in the samples**. Note the logarithmic scales on both axes.

**Figure 3:**
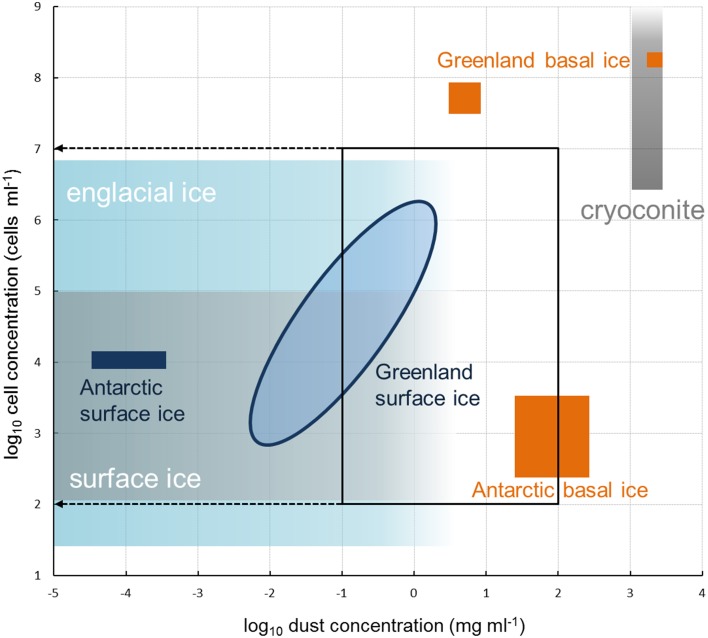
**Microbial cell abundances and dust concentrations in glacier ice samples**. Blue oval represents samples collected in this study and measured by EFM (see Figure 2); the remainder of the data was compiled from the literature (see Table 4). Black frame represents the ranges used for method testing in this study. Note that due to logarithmic scales on both axes zeroes cannot be shown.

The abundances (10^3^–10^6^ cells per ml of melted ice) and dust concentrations (0.01–2 mg ml^−1^) determined in surface ice samples in this study (Tables 2, 3; Figure 2) fit within the ranges reported for glacier ice samples from around the globe (Figure 3). Table 4 shows an overview of the published data of cell abundances and dust concentrations in various glacier samples, including glacier snow and clean englacial ice with little dust and few cells, microbe-rich surface ice and debris slurries, and debris-laden basal ice with widely ranging cell abundances. These differences suggest a role of particulates for microbial abundance, which is further supported by the rich microbial community associated with cryoconite, where microbial abundance may reach 10^6^–10^9^ cells g^−1^, as determined by EFM (Stibal et al., 2008a, 2010, 2012b; Anesio et al., 2010; Hodson et al., 2010a; Langford et al., 2010) and qPCR (Hamilton et al., 2013; Zarsky et al., 2013; Stibal et al., 2015).

**Table 4:** **Microbial cell abundances and dust concentrations in glacier snow, ice, and ice/debris mixture samples**.

**Sample type**	**Location**	**Cell count method**	**Cell abundance (10^3^ ml^−1^)**	**Dust/debris concentration (mg ml^−1^)**	**References**
Supraglacial snow	Greenland	EFM	2.4–15^*^	0.37^*^	This study
	Antarctica	EFM	0.2–5	n.d.	Carpenter et al., 2000
	Svalbard	EFM	0.03–40	n.d.	Amato et al., 2007; Björkman et al., 2014
	Central Asia	FCM	0.68–720	n.d.	Liu et al., 2009
Surface ice	Greenland	EFM	1.9–1900	0.01–1.87	This study
		FCM	0–71		
		qPCR	2.4–2600^**^		
	Svalbard	EFM	200	n.d.	Amato et al., 2007
		FCM	57	n.d.	Irvine-Fynn et al., 2012
Englacial ice	Greenland	FCM	20–7000	0–0.005	Svensson et al., 2000; Tung et al., 2005; Miteva et al., 2009
	Antarctica	EFM	0.2–36	0–0.005	Karl et al., 1999; Priscu et al., 1999; Abyzov et al., 2001; Antony et al., 2012
	Central Asia	EFM	0.02–170	n.d.	Zhang et al., 2008a,b
		FCM	3.2–830	n.d.	Yao et al., 2008; An et al., 2010
Cryoconite hole ice/water	Antarctica	EFM	0.26–79	n.d.	Foreman et al., 2007; Hodson et al., 2013
	Svalbard	EFM	4.5–100	n.d.	Säwström et al., 2002; Mindl et al., 2007; Anesio et al., 2010
Cryoconite slurry	Antarctica	EFM	40–3800	n.d.	Foreman et al., 2007; Hodson et al., 2013
Basal ice	Greenland	EFM	6–30 × 10^4^	up to ~1600	Sheridan et al., 2003; Yde et al., 2010
	Antarctica	EFM	0.1–4.2	20–280	Montross et al., 2014

*n.d., not determined*.

* *Data from the Saddle ice core 2013 winter snow layer (18–42 cm depth)*.

** *Assuming 10 16S rRNA gene copies per cell*.

**Figure 4:**
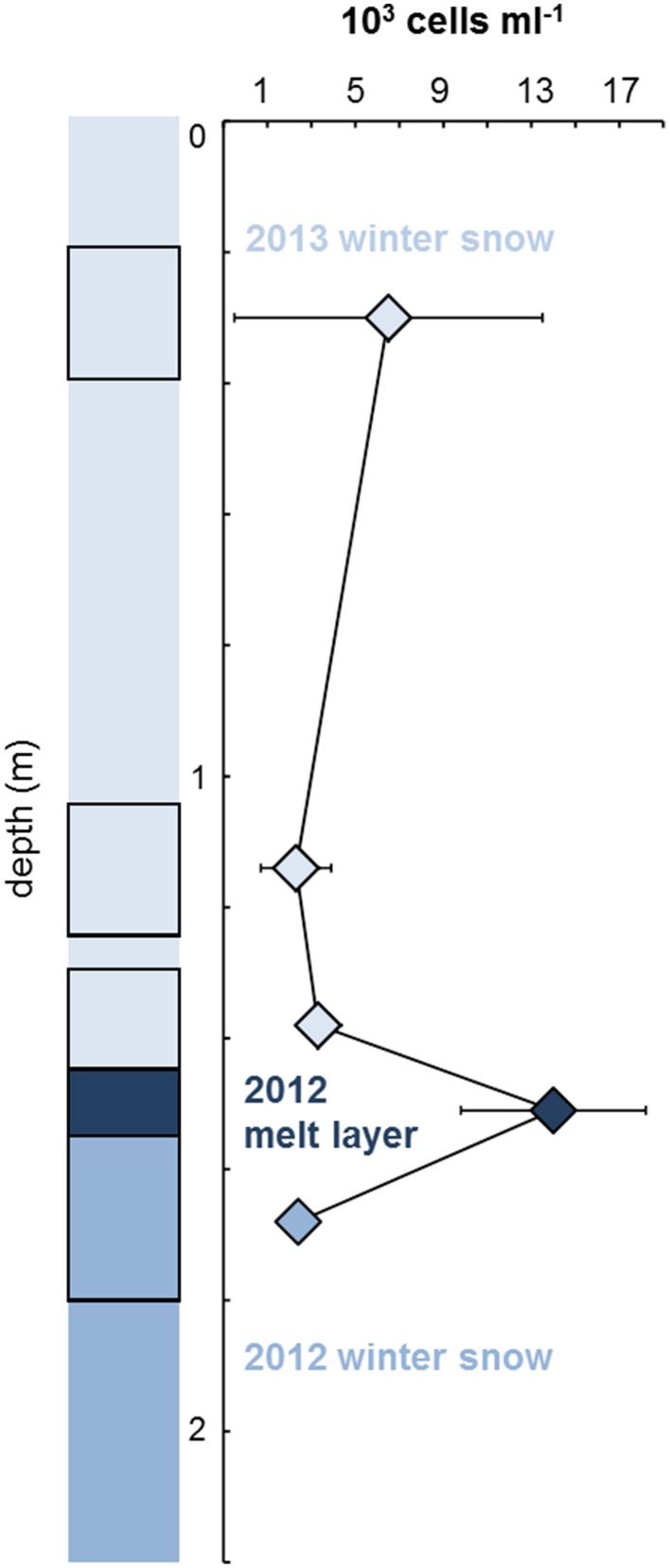
**Cell abundance measured by EFM in the Saddle ice core**. The five sections used for enumeration are depicted by black frames. Values are means ± st.devs. of three measurements (2013 winter snow top layer, 2012 melt layer) or two measurements (remaining samples).

Figure 4 illustrates microbial abundances measured by EFM in five sections of the 2.2 m deep Saddle firn core, representing winter snow from 2013 (18–42, 105–123, 130–147 cm) and 2012 (157–180 cm) and the 2012 summer melt layer between them at 147–157 cm depth. The abundance of cells in the 2012 summer melt layer (14,000 cells ml^−1^) was an order of magnitude higher than the other analyzed core samples, especially in comparison with the immediately underlying and overlying snow layers (2400 and 3300 cells ml^−1^, respectively). It should be noted however that, due to the small amount of sample volume available for analysis and the expected low cell concentrations, few replicates were measured and the differences are thus not significant. The abundances fall in the range reported from snow on glaciers on the Tibetan Plateau (0.7–700 × 10^3^ cells ml^−1^; Liu et al., 2009) and on the Antarctic ice sheet (200–5000 cells ml^−1^; Carpenter et al., 2000), and are somewhat lower that those found in Svalbard glacier snow (10–40 × 10^3^ cells ml^−1^; Amato et al., 2007; Table 4). They are also similar to cell abundances determined by EFM in snow over sea ice in NE Greenland (0.8–3 × 10^3^ cells ml^−1^; Møller et al., 2013). The elevated abundance detected in the 2012 melt layer (Figure 4) may be a result of microbial growth during the short melt event in July 2012 (Nghiem et al., 2012), as suggested by Hell et al. (2013), and could represent a glimpse into the warmer future of the ice sheet; however, more data are needed to test this hypothesis.

### Controls of microbial abundance in surface ice on the GrIS

In order to explain the variation in the microbial abundance data, a RDA was performed with physico-chemical data (position along the N-S transect expressed as the N coordinate, altitude, distance from the margin, surface type, days with positive surface air temperature, days since last snowfall, and day of sampling from Table 1; dust content, EC, pH, and nutrient concentrations from Table 2) as the explanatory variables, and microbial abundance data (Table 3) as the explained variables. Several analyses were performed; first, with all the data available, and, subsequently, with some data removed due to their suspected lower accuracy. The APO sample data were removed due to their potential contamination, and the FCM abundance data were removed due to the low accuracy and reproducibility shown in the artificial ice experiments (Supplementary Figure S1). Data from the Saddle ice core could not be used due to the absence of the qPCR and FCM data and most physico–chemical data. The removal of the FCM abundance data and APO samples from the analysis resulted in a higher amount of total variability explained (data not shown).

**Figure 5:**
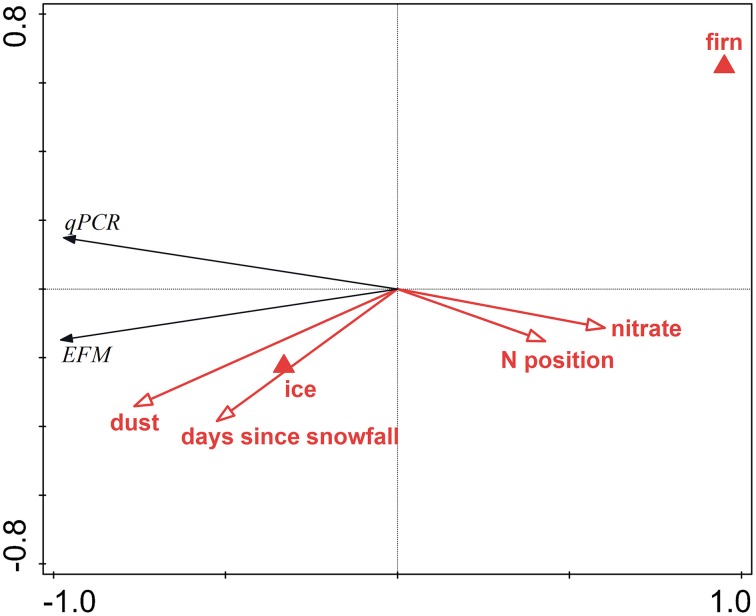
**Redundancy analysis biplot visualizing the effects of environmental variables on the microbial abundance in surface ice on the GrIS**. Red arrows denote significant quantitative physical variables, red triangles the surface type, and black arrows the abundances determined by EFM and qPCR.

Analysis that ignored FCM data and APO samples explained 97.3% of the total variation in the data. Dust content was the most significant variable, explaining 55.9% of the variation (pseudo*F* = 36.7; *p* = 0.006), followed by surface type (ice vs. firn; 14.6% explained, pseudo*F* = 13.9, *p* = 0.012), nitrate concentration (6.7% explained, pseudo*F* = 7.9, *p* = 0.027), and days since last snowfall (5.2% explained, pseudo*F* = 37.3, *p* = 0.006). Although the day of sampling was not a significant factor in this analysis (pseudo*F* = 2.7, *p* = 0.20), it is essentially an artifact of the sampling design, and, therefore, another RDA was conducted with this parameter as a covariate, thus showing only the results for the ecologically meaningful variables. This analysis explained 96.2% of the total variation; dust content explained 41.8% of the variation (pseudo*F* = 20.1; *p* = 0.004), followed by surface type (20.1% explained, pseudo*F* = 13.4, *p* = 0.007), the N-position (10.8% explained, pseudo*F* = 10.1, *p* = 0.019), and days since last snowfall (7.5% explained, pseudo*F* = 37.3, *p* = 0.006). Figure 5 is an RDA biplot that illustrates the positive correlations of microbial abundance and dust contents and days since last snowfall, the negative correlation between cell numbers and the N-coordinate, and the preference of microbial cells for ice compared with firn. The relationship between dust content and cell numbers in surface ice on the GrIS is also illustrated in Figure 2 in which the EFM abundance data are plotted against dust contents in all samples including those from APO and Saddle, showing a positive correlation between dust and cell numbers (*R*^2^ = 0.89 with all data used; *R*^2^ = 0.81 with APO samples removed).

The variation in microbial abundance in the surface ice samples collected on the GrIS reflects the differences between the sites and the important effect of local conditions on biological processes in the supraglacial ecosystem. The lowest abundances in our study (~10^3^ cells ml^−1^) were found in samples from the accumulation area of the ice sheet (KAN_U, Saddle) or in those affected by fresh snow (KAN_L), and are similar to abundances found in atmospheric waters (Sattler et al., 2001; Bowers et al., 2012). Since microbial cells may act as ice nuclei (Christner et al., 2008; Delort et al., 2010), the lowest abundances found in surface ice may represent a “baseline” cell concentration, which is a result of deposition of snow already containing microbial cells.

Dust deposition is another possible source of microbial cells to the ice sheet (Xiang et al., 2009). Simultaneous analysis of dust and cell concentrations from glacial ice samples is scarce (e.g., Antony et al., 2012), and some studies suggest that microbial abundance in glacial ice cores is not always associated with dust deposition (Zhang et al., 2008a; Xiang et al., 2009). However, the results of the statistical analysis of our data show for the first time a significant association between dust and cell abundance in Greenland surface ice (Figures 2, 5). This strong correlation may be explained in two ways: first, microbial cells may be deposited onto the GrIS in association with dust particles, and second, dust may provide a source of nutrients to stimulate the growth of microbes in the vicinity. Phosphorus, a rock-bound nutrient, is likely the limiting macronutrient in the supraglacial environment (Stibal et al., 2008b, 2009) and has been detected in surface debris on the southwestern GrIS (Wientjes et al., 2011), which supports this hypothesis.

Microbial abundance was also shown to be correlated to surface type (with ice showing higher cell numbers than firn) and the number of days since the last snowfall (Figure 5). We suggest that these controls are related to the process of cell retention at the glacier surface. This process begins in melting snow (Hell et al., 2013; Björkman et al., 2014) and continues in surface ice, which potentially acts as a filter (Irvine-Fynn et al., 2012). Therefore, the bare ice surface is expected to accumulate more microbial cells over time compared with firn, unless their abundance is “diluted” by fresh snow. The preference of ice over firn can also be explained by the longer melt period at the sites with ice compared to those with firn, which is further supported by the significant effect of the N-S position, and is also likely related to the length of the melt season. Difference in the amount of solar radiation is another possible explanation of the significance of the N-S position. The significant negative correlation between microbial abundance and nitrate concentration could be interpreted as a result of microbial uptake of nitrate (Telling et al., 2012) and thus a sign of an active microbial community in surface ice on the ice sheet.

## Conclusions

We quantified for the first time the abundance of microbial cells in surface ice from geographically distinct sites on the GrIS, including ablation and accumulation areas, using three different methods (EFM, FCM, and qPCR). EFM generated the most accurate and reproducible results of the three methods, and is therefore recommended for the cell enumeration of glacier ice. Cell abundance of surface ice samples, determined by EFM, ranged from ~2 × 10^3^ to ~2 × 10^6^ cells ml^−1^, while the dust concentrations were found to be between 0.01 and 2 mg ml^−1^. Dust content was the most significant factor explaining the variation in abundance data. Surface type (ice vs. firn), number of days since last snowfall, N-S position and nitrate concentration were also identified as significant controls. We suggest that the surface of the Greenland Ice Sheet receives a “baseline” cell supply via deposition of atmospheric waters, and that wind-borne dust deposited on the ice sheet likely contains additional cells and may provide limiting nutrients for microbial growth. Ablation areas with high dust concentrations and longer melt seasons are therefore expected to contain higher numbers of active microbes compared to the accumulation area and those portions of the ablation area that contain little dust and are primarily seeded with atmospheric waters.

## Author contributions

MS conceived and designed the study with inputs from JB, EG and CJ; MS, MS, JZ and JB collected samples; EG prepared the artificial ice samples and performed microscopy and qPCR assisted by MS, KC and CJ; AE, IS, JG and TI did flow cytometry analyses; JB provided glaciological and climate data for sampling sites; all authors contributed to the discussion of the results; MS wrote the paper with inputs from EG, KC, JB, IS, AE, TI, and CJ.

### Conflict of interest statement

The authors declare that the research was conducted in the absence of any commercial or financial relationships that could be construed as a potential conflict of interest.

## Supplementary material

Supplementary Figure S1 **Accuracies and standard deviations of cell abundance measurements of artificial ice samples by epifluorescence microscopy (EFM), flow cytometry (FCM) and quantitative PCR (qPCR)**. Note the logarithmic scales and differences in color scaling between plots.
